# 2D Quantum Spin-Liquid Candidate Including a Chiral Anion: κ-(BEDT-TTF)_2_[B_*R*/*S*_(salicylate)_2_]

**DOI:** 10.1021/jacs.4c12386

**Published:** 2025-02-06

**Authors:** Toby J. Blundell, Kathryn Sneade, Joseph O. Ogar, Satoshi Yamashita, Hiroki Akutsu, Yasuhiro Nakazawa, Takashi Yamamoto, Lee Martin

**Affiliations:** † School of Science and Technology, Nottingham Trent University, Clifton Lane, Clifton, Nottingham NG11 8NS, U.K.; ‡ Department of Chemistry, Graduate School of Science, Osaka University, 1-1 Machikaneyama-cho, Toyonaka, Osaka 560-0043, Japan; § Graduate School of Science and Engineering, Ehime University, Matsuyama 790-8577, Japan; ∥ Geodynamics Research Center, Ehime University, Matsuyama 790-8577, Japan

## Abstract

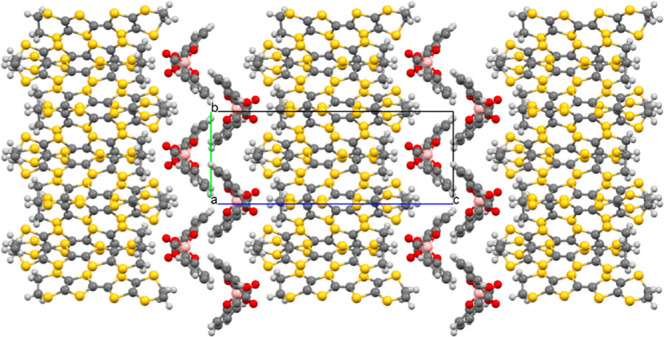

The quantum spin-liquid state was first theorized by Anderson 50 years ago and the challenge remains to realize a quantum spin-liquid material. A handful of two-dimensional molecular candidates have attracted huge attention over the past 30 years owing to their triangular lattice possessing *S* = 1/2 spin systems. We present a new quantum spin-liquid candidate in 2D Mott insulator κ-(BEDT-TTF)_2_[B_*R*/*S*_(salicylate)]_2_. The structure has a double-width anion layer giving it a strong 2D character. The spiroborate anion is chiral and the salt is an inversion twin, having no inversion symmetry center and crystallizing in space group *P*2_1_. This offers the possibility of novel behavior owing to the low symmetry not previously seen in molecular spin-liquid candidates. The peak height of the 6K anomaly of κ-(BEDT-TTF)_2_[B_*R*/*S*_(salicylate)_2_] is 2 or 3 times larger than that of κ-(BEDT-TTF)_2_Cu_2_(CN)_3_ and EtMe_3_Sb[Pd(dmit)_2_]_2_. The structure presents many opportunities for crystal engineering through atom-by-atom changes to the ligands on the spiroborate anion to produce a family of materials which lie in and around the QSL region of the phase diagram for these salts. This gives the prospect of an experimental playground to deepen understanding of the QSL state. Electrical resistivity, SQUID magnetometry, band calculations, heat capacity, Infrared and Raman spectroscopy are reported for κ-(BEDT-TTF)_2_[B_*R*/*S*_(salicylate)_2_].

## Introduction

Chirality, where a molecule or material can exist as two mirror image forms which are nonsuperimposable, is ubiquitous and living organisms rely on chiral molecules, such as amino acids, proteins, nucleic acids, and sugars. The question of whether chirality has an effect on the electrical and magnetic properties of a material has only recently been experimentally observed owing to a lack of enantiopure materials in nature. Molecular conductors offer the possibility of introducing chirality into a multifunctional inorganic–organic material and tuning the physical properties through structural alterations to the molecules.

Electrical magneto-chiral anisotropy (eMChA) has been observed in bismuth helices^1^ and carbon nanotubes.^2^ In the presence of an applied magnetic field, differences are observed in the electrical resistivity of the opposing chiral enantiomers of a material. This eMChA effect is greatly enhanced when a noncentrosymmetric material (WS_2_^3^ or MoS_2_^4^) enters the superconducting state, with a difference in electrical resistivity being observed depending on the direction of the electric current in an applied magnetic field. The adsorption of a monolayer of chiral molecules onto the surface of a conventional superconductor has also been shown to produce a marked change in the diamagnetic Meissner response.^5^ Organic molecular conductors offer an advantage over the aforementioned inorganic conductors because they can be synthesized with stereogenic centers and obtained in both enantiomeric forms, and also as the racemate, to allow a direct comparison of their properties. One such example showing eMChA are (*S*,*S*)- and (*R*,*R*)-(DM-EDT-TTF)_2_ClO_4_^6^ which crystallize in enantiomorphic chiral space groups *P*6_2_22 and *P*6_4_22, and show metallic behavior down to 40 K. An organic molecular conductor of enantiopure donor molecule (1′*R*,5*S*)-*N*-(1′-phenylethyl)(BEDT-TTF)acetamide with TCNQ remains metallic down to 4.2 K.^7^

Another property of interest in chiral molecular conductors is the chirality-induced spin selectivity (CISS) effect which has implications over many fields including enantioseparation, chiral spintronics, and electron-transfer processes.^8^ Electrons of a certain spin can traverse the material more easily depending on the handedness of the chiral material owing to the CISS effect. The CISS effect has recently been observed in a molecular superconductor which crystallizes in space group *P*2_1_, owing to the handedness of the relative arrangement of anion and cation.^9^

Previously we have reported organic molecular conductors of BEDT-TTF with spiroborates^10,11^ where only a certain enantiomer/diastereomer of the anion is included in the radical-cation salt despite there being a mixture of enantiomers/diastereomers in solution during synthesis. In some cases when using a chiral spiroborate anion starting material the crystals obtained with BEDT-TTF are helical shaped.^10,11^ A chiral organic molecular conductor of BDH-TTP, with the enantiopure [B(2-chloromandelate)_2_]^−^ anion is a metal down to at least 4.2 K.^12^

Only a handful of quantum spin-liquid candidate (QSL) materials have previously been reported in organic molecular dimer Mott insulators such as κ-(BEDT-TTF)_2_Cu_2_(CN)_3_,^13^ κ-(BEDT-TTF)_2_Ag_2_(CN)_3_,^14^ EtMe_3_Sb[Pd(dmit)_2_]_2_,^15^ κ-H_3_(Cat-EDT-TTF)_2_.^16^ These have proven to be prime examples for study of the QSL state which Anderson first theorized 50 years ago.^17^ Electron spins in magnetic materials in a square grid can align with their neighboring electrons to give long-range ordered magnetic behavior, such as ferromagnetism or antiferromagnetism, at lower temperatures. The QSL state arises from a triangular network where electron spins cannot all align with their neighbors resulting in magnetic frustration which suppresses long-range magnetic order even at very low temperatures. The organic radical-cation salts which are QSL candidates have a triangular lattice of (BEDT-TTF)_2_ dimers, and of these, κ-(BEDT-TTF)_2_Cu_2_(CN)_3_,^13^ having a close to ideal equilateral triangular lattice, has been the most studied. Here we report a new QSL candidate in a κ-type BEDT-TTF radical-cation salt with the spiroborate [B_*R*/*S*_(salicylate)_2_]^−^ anion which is a Mott insulator. This salt is also highly two-dimensional, having a double anion layer segregating each conducting BEDT-TTF donor layer. The inclusion of the chiral spiroborate anion leads to this material having no inversion symmetry center, crystallizing in space group *P*2_1_. This gives the possibility of novel behavior owing to the low symmetry not previously seen in molecular QSL candidates. These materials would be expected to exhibit the chirality-induced spin selectivity effect, and possibly spin valve behavior. Moreover, in systems containing racemic mixtures of both enantiomers, smaller structural distortions can be introduced compared to impurity doping or mixed alloys, potentially enhancing physical properties associated with quantum critical points. Therefore, the introduction of chiral spiroborate anions offers a valuable opportunity to explore new frontiers in quantum materials.

## Results and Discussion

### Crystal Structure of κ-(BEDT-TTF)_2_[B_*R*/*S*_(salicylate)_2_]

κ-(BEDT-TTF)_2_[B_*R*/*S*_(salicylate)_2_] crystallizes in the noncentrosymmetric space group *P*2_1_ with the asymmetric unit containing two independent BEDT-TTF molecules and a single molecule of the spiroborate anion (Figure S1). There is only a single enantiomer of the [B_*R*/*S*_(salicylate)_2_]]^−^ anion present, despite the spiroborate starting material in solution being a labile racemic mixture of B_*S*_ and B_*R*_ and there being no chirality on the salicylate ligands (Figure S2). Spontaneous resolution was previously observed for isostructural chiral metal κ-(BDH-TTP)_2_[B_*S*_(*S*-ClMan)_2_],^12^ and attributed to the differences in shape between [B_*S*_(*S*-ClMan)_2_] which is V-shaped, and [B_*R*_(*S*-ClMan)_2_] which is twisted.^18^ κ-(BEDT-TTF)_2_[B_*R*/*S*_(salicylate)_2_] has a Flack parameter of 0.52(6), making it an inversion twin. This indicates that homogeneous domains within the crystal each consist of only a single enantiomer of either B_*S*_ or B_*R*_, with B_*S*_ domains being the mirror image of the B_*R*_. The B_*S*_ enantiomer of the salicylate spiroborate anion has previously been resolved in the solid state in high yield and purity by metathesis of Na[B_*S*_(salicylate)_2_] with quinine.^19^ The opposing enantiomer [B_*R*_(salicylate)_2_] has also been resolved via crystallization with sparteine with 80% ee, while the [B_*R*_(5-chlorosalicylate)_2_] can be obtained with 100% ee.^19^

**Figure 1:**
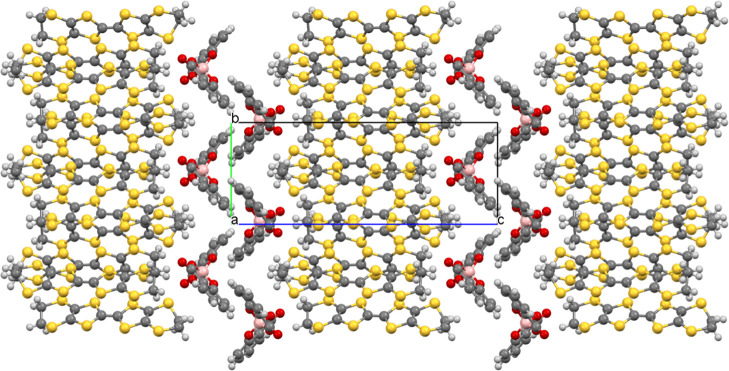
Layered structure of κ-(BEDT-TTF)_2_[B_*R/S*_(salicylate)_2_] viewed down the *a* axis.

**Figure 2:**
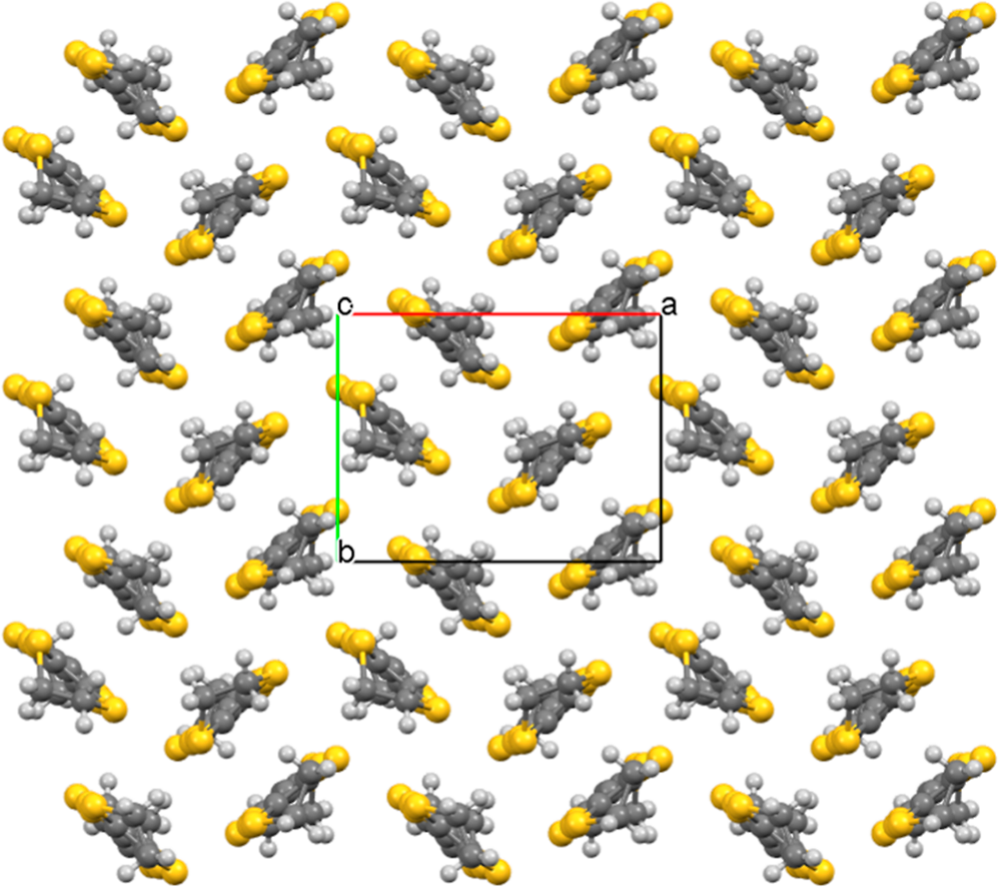
Donor packing of κ-(BEDT-TTF)_2_[B_*R/S*_(salicylate)_2_] viewed down the *c* axis.

The BEDT-TTF molecules in κ-(BEDT-TTF)_2_[B_*R*/*S*_(salicylate)_2_] pack in the *ab* plane with donor stacks separated in the *c* direction by a double layer of [B_*R*/*S*_(salicylate)_2_]^−^ anions (Figures 1 and S3). The BEDT-TTF molecules form a κ-type packing (Figure 2) with a number of S···S contacts present between BEDT-TTF molecules (Table S1). As is common for κ-type packing there are no direct short contacts between the face-to-face donor pairs. Each BEDT-TTF dimer pair accommodates spin *S* = 1/2, with the dimers arranged in a triangular packing arrangement.

The molecular formula suggests that the two independent donors have a cumulative charge of +1 to balance the charge of the [B_*R*/*S*_(salicylate)_2_]^−^ anion.

The spiroborate anions form a double layer (Figure 1). A single anion layer is shown in Figure S4. This double anion layer has a width of ∼9.5 Å—being the shortest distance between terminal ethylene groups of BEDT-TTF of adjacent donor stacks, compared to the ∼4.6 Å in QSL candidate κ-(BEDT-TTF)_2_Cu_2_(CN)_3_ (CCDC 1016296).

### Conducting Properties

**Figure 3:**
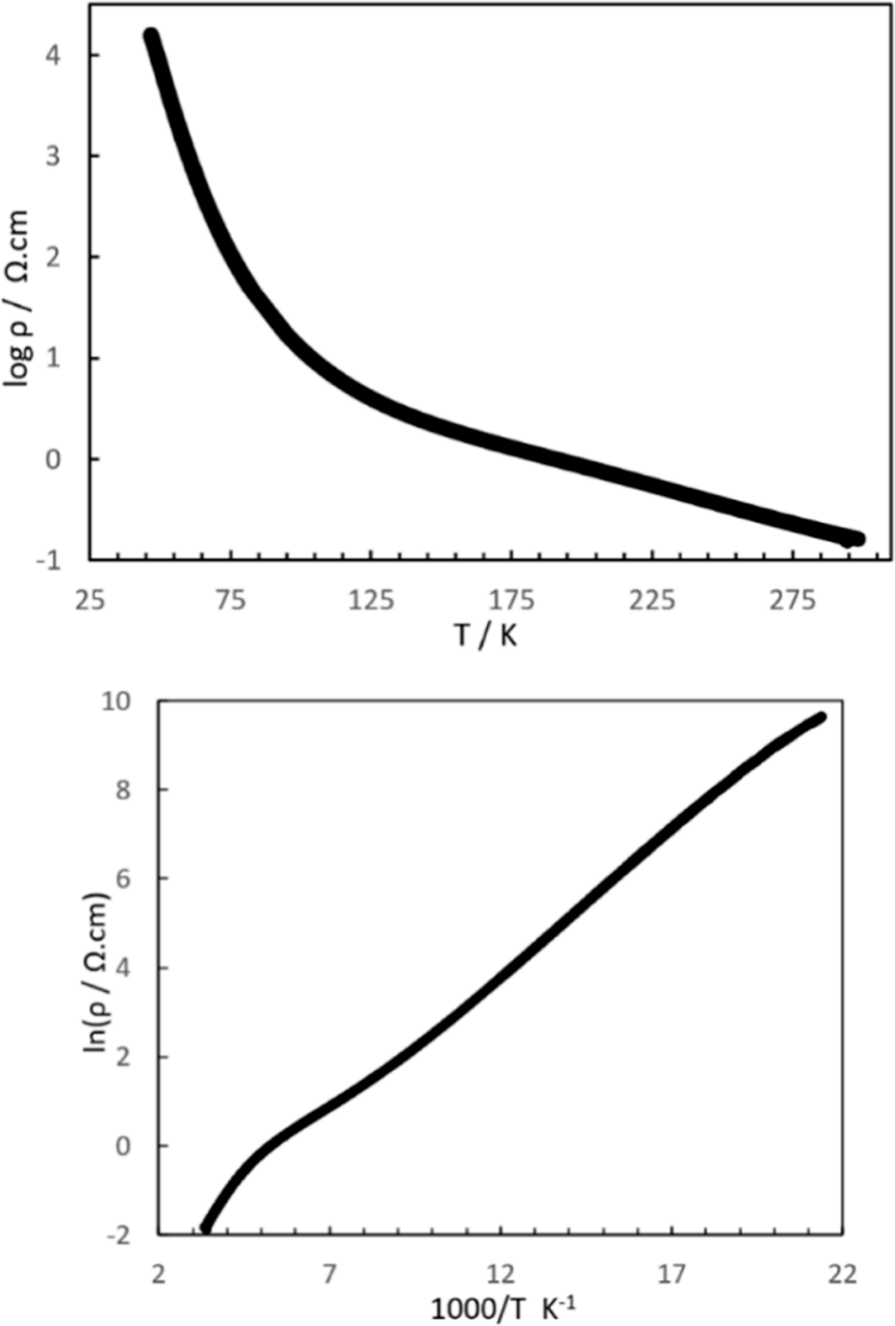
Electrical resistivity for κ-(BEDT-TTF)_2_[B_*R/S*_(salicylate)_2_].

**Figure 4:**
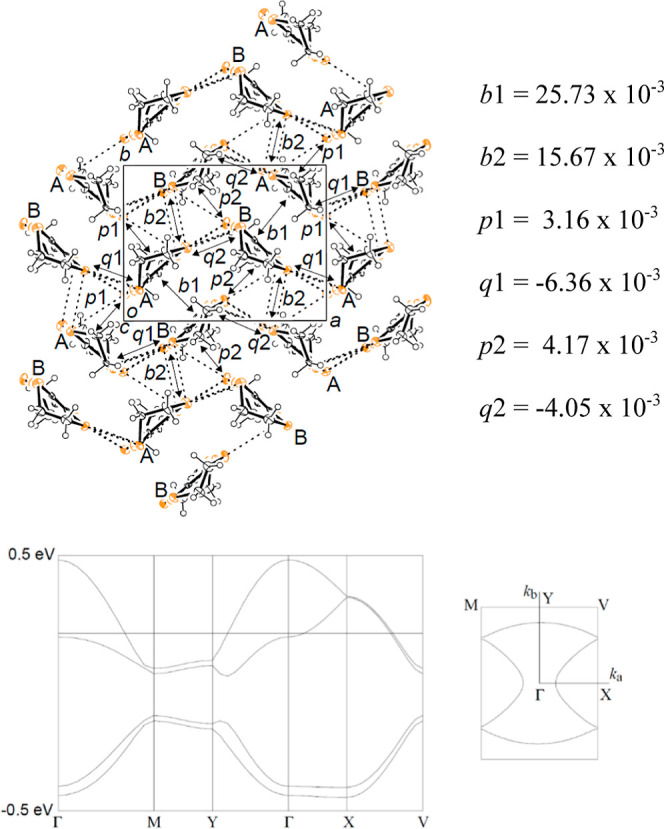
Band calculation for κ-(BEDT-TTF)_2_[B_*R/S*_(salicylate)_2_].

Figure 3 shows the temperature dependence of the electrical resistivity. Semiconducting behavior is observed with an activation energy of 0.13 eV indicating that κ-(BEDT-TTF)_2_[B_*R*/*S*_(salicylate)_2_] can be categorized as a dimer Mott insulator. This is comparable to the 0.12 eV observed for κ-(BEDT-TTF)_2_Cu_2_(CN)_3_.^13^ Band structure calculation has been performed using the extended Hückel method^20^ and shows κ-like Fermi surfaces (Figure 4).

The band structure obtained using parameters commonly employed in calculations for BEDT-TTF salts tends to predict metallic conductivity; however, since these calculations ignore electron correlations, they fail to predict semiconductor-like behavior. Indeed, the infrared spectra exhibit mid-infrared transitions indicative of electron correlation effects, suggesting that the band structure is not actually metallic.

The triangular lattice of BEDT-TTF dimers in κ-(BEDT-TTF)_2_[B_*R*/*S*_(salicylate)_2_] has two different triangles because of the *P*2_1_ space group. The frustration parameter *t*′/*t* of the triangles was calculated according to the literature^21^ as *t*′/*t*1 = 1.65 (*p*1, *q*1) and *t*′/*t*2 = 1.91 (*p*2, *q*2) (average = 1.77). These values are much higher than in other QSL candidates where a value closer to unity for an equilateral triangle and a fully frustrated system is observed for κ-(BEDT-TTF)_2_Cu_2_(CN)_3_ (*t*′/*t* = 0.83)^22^ or EtMe_3_Sb[Pd(dmit)_2_]_2_ (*t*′/*t* = 0.92).^23^ The situation here is similar to that in κ-(BEDT-TTF)_2_TaPF_6_ (*t*′/*t* = 1.76)^21^ which demonstrates that an equilateral triangle is not a requirement for the spin-liquid state.

### Magnetic Properties

**Figure 5:**
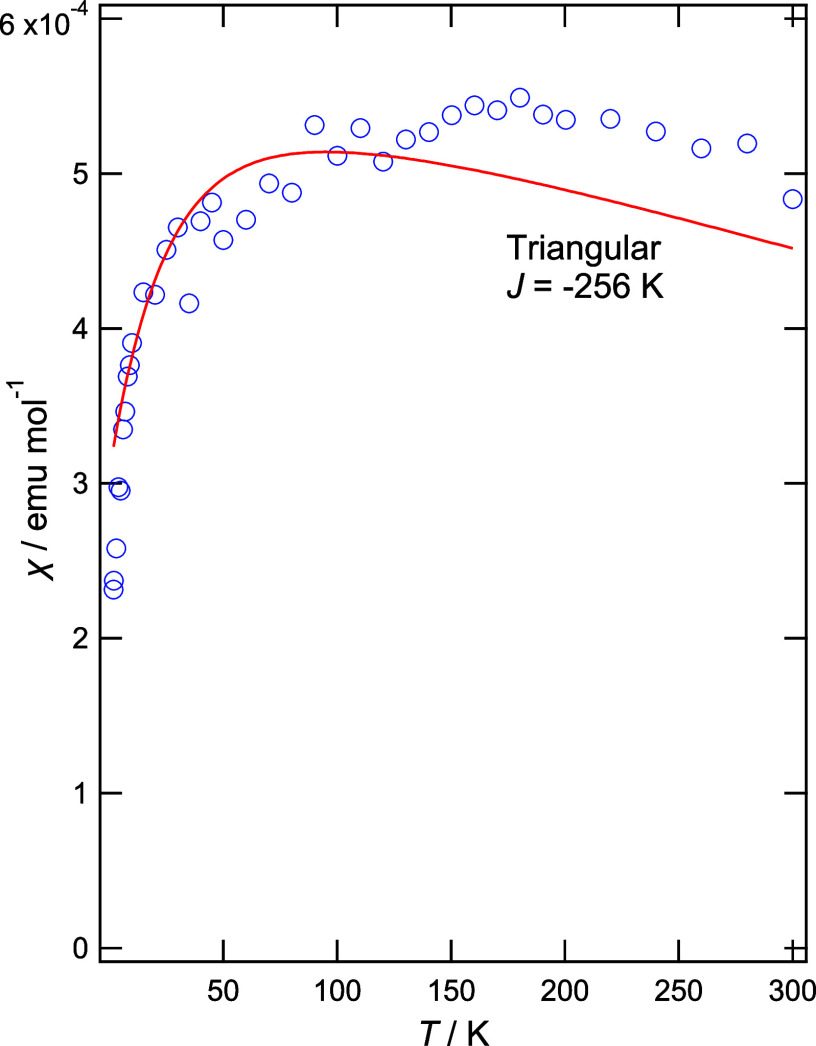
Magnetic susceptibility for κ-(BEDT-TTF)_2_[B_*R/S*_(salicylate)_2_].

SQUID magnetometry was performed on a polycrystalline sample and Figure 5 shows the spin susceptibility fitted to a 2D spin 1/2 antiferromagnetic Heisenberg triangular model giving a rough estimation of coupling constant *J* = –256 K, which is comparable to the ∼250 K found in κ-(BEDT-TTF)_2_Cu_2_(CN)_3_^22^ and the 220–250 K found in EtMe_3_Sb[Pd(dmit)_2_]_2_.^24^

### Infrared and Raman Spectroscopy

**Figure 6:**
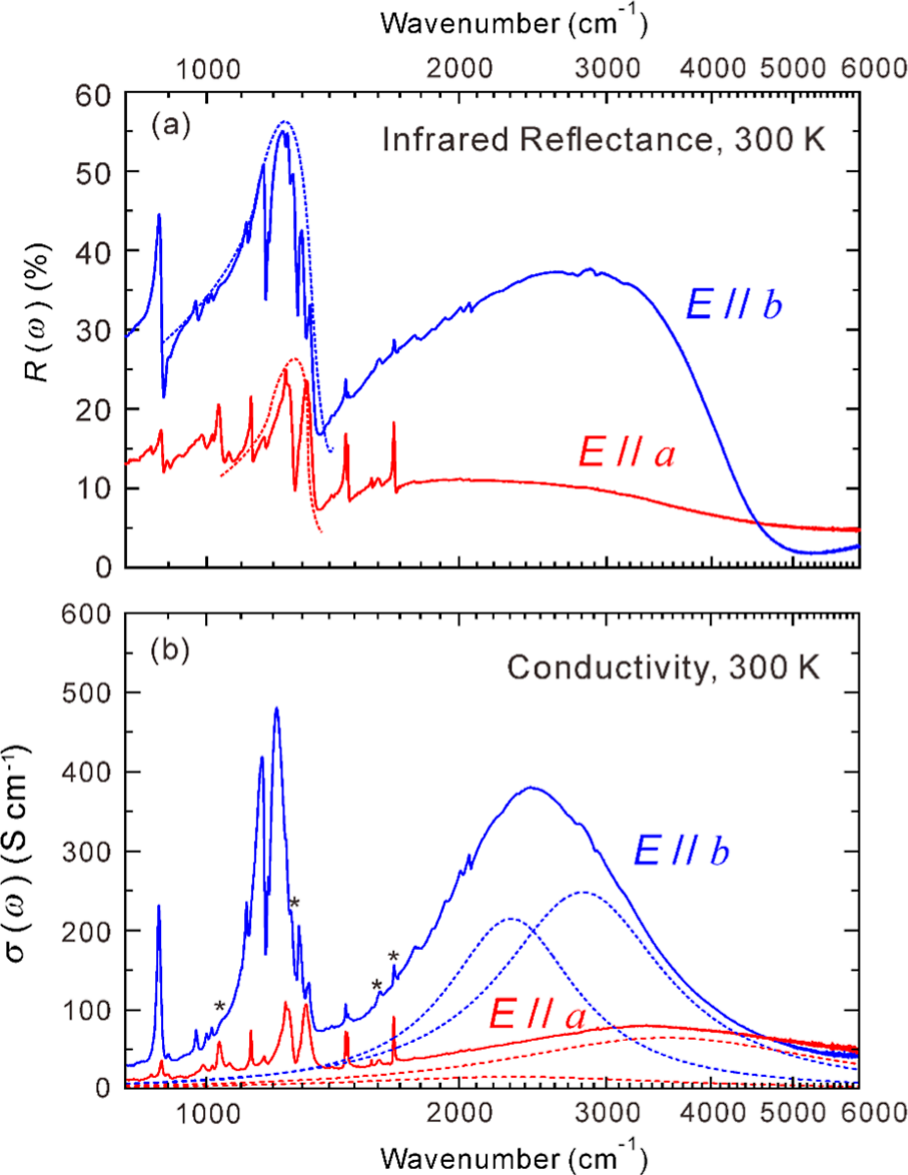
(a) Polarized spectra of the infrared reflectance *R*(ω) for κ-(BEDT-TTF)_2_[B_R/S_(salicylate)_2_]. Dashed lines in the *a*-polarized spectra denote either ν_3–3_ or ν_3–4_ modes defined in Figure S5, while those in the *b*-polarized spectra denote the rest. (b) Polarized conductivity spectra obtained from the reflectance spectra. Red and blue dashed lines at 3540 and 2810 cm^–1^, respectively, denote the MIR transitions originating from *t*_d_, while those at 2320 and 2310 cm^–1^ denote the MIR transitions originating from *t*′ and *t*.

Figure 6a shows the polarized spectra of the infrared reflectance *R*(ω) measured at 300 K. Figure 6b presents the polarized conductivity spectrum σ(ω) obtained by Kramers–Kronig transformation of the infrared spectrum. The reflectance decreases from 1000 to 800 cm^–1^ for both polarization directions. This suggests semiconducting behavior rather than the Drude type characteristic of metallic materials; this is in fact consistent with the temperature dependence of the electrical resistance. Similarly, the conductivity spectrum has a baseline below 1000 cm^–1^ that is almost zero, except for the sharp peaks of intramolecular vibrations.

Between 800 and 4500 cm^–1^, both the reflectance and conductivity of the *b*-polarized spectrum are greater than those of the *a*-polarized spectrum. The peaks at approximately 2440 cm^–1^ in the *b*-polarized conductivity spectrum and around 3300 cm^–1^ in the *a*-polarized conductivity spectrum are mid-infrared (MIR) transitions proportional to the magnitudes of the transfer integrals between the dimers, *t*′ and *t* as well as the intradimer transfer integral (*t*_d_, which is proportional to *b*_1_).^25^ The intensity of *b* polarization is greater than that of *a* polarization. This polarization dependence is opposite to the behavior of most κ-type BEDT-TTF salts.^25−30^ The same polarization dependence as κ-(BEDT-TTF)_2_B(CN)_4_ indicates that *t*′ is larger than *t*.^31^ In general the absolute values of the transfer integrals derived from reflectivity measurements do not agree with those calculated from X-ray structural analysis.^30,32^ Nevertheless, the ratio of *t*′ to *t* estimated from both method has been successfully validated in previous studies using κ-(BEDT-TTF)_2_B(CN)_4_.^31^ Therefore, we will perform a validation of the ratio rather than absolute value here. The ratio *I*_b_/*I*_a_ of the integrated intensity of conductivity in the region between 1550 and 6000 cm^–1^, where no strong intramolecular vibrations are observed, is related to the ratio *t*′/*t* of the transfer integral, with *I*_b_/*I*_a_ = 1.3 × *t*′/*t*.^31^ In κ-(BEDT-TTF)_2_[B_*R*/*S*_(salicylate)_2_], *I*_b_/*I*_a_ = 7/3, which gives *t*′/*t* = 1.8. This value is in very good agreement with the averaged *t*′/*t* = 1.77 obtained from calculations based on X-ray structure analysis results. This value is larger than the *t*′/*t* value of 1.5 for κ-(BEDT-TTF)_2_B(CN)_4_ at the same temperature of 300 K.^31^ Therefore, it is clear that the 2D conducting layer of κ-(BEDT-TTF)_2_[B_*R*/*S*_(salicylate)_2_] is more one-dimensional than that of κ-(BEDT-TTF)_2_B(CN)_4_.

However, the broad peak observed between 1550 and 6000 cm^–1^ is not attributed to a single type of mid-infrared (MIR) transition but rather consists of two distinct types.^25^ It should be noted that the effect of on-site coulomb repulsion within an ET monomer (*U*_ET_, from 6000 to 7000 cm^–1^) is negligible in the region shown in Figure 6.^33^ One type is a transition related to the coulomb repulsion between neighboring intradimer molecules (*V*_d_), often referred to as the on-site coulomb repulsion within a dimer (*U*_d_). The other type is a transition associated with the average coulomb repulsion between neighboring interdimer molecules (*V*_inter_). The former is proportional to *t*_d_, while the latter is proportional to *t*′ and *t*, as both intersite coulomb repulsion and transfer integrals depend on the interplanar distance, dihedral angle, and slipping distance between neighboring molecules.^34^

The former shows polarization dependence and tends to occur between 2500 and 4000 cm^–1^ in the *b*- and *a*-polarized spectra, respectively, while the latter appears around the 2000 cm^–1^ region. A similar result was also observed in κ-(BEDT-TTF)_2_B(CN)_4_.^31^ To rigorously evaluate the anisotropy, it is preferable to focus exclusively on the MIR transition anisotropy influenced by *t*′ and *t*, while minimizing the effects of *t*_d_. Curve fitting, assuming multiple Lorentzian contributions to the dielectric function, applied to the reflectance spectra, is necessary to reduce the influence of *t*_d_ as much as possible. We performed curve fitting for each polarization direction, incorporating the dominant intramolecular vibrations. The two types of MIR transitions are shown as dashed lines in Figure 6b. In the *b*-polarized and *a*-polarized spectra, the low-wavenumber MIR transition positions are nearly identical, at 2310 and 2320 cm^–1^, respectively. The area ratio of these peaks is 2.34, which closely matches the ratio including both MIR transitions *I*_b_/*I*_a_ = 7/3 = 2.33. The consistency between the results obtained with and without *t*_d_ indicates that the ratio of the *b*- and *a*-components of *t*_d_ is nearly identical to the ratio of the *b*- and *a*-components of the interdimer transfer integrals.

From the average peak positions obtained from the fitting results of the *a*- and *b*-polarized spectra, rough estimates for *V*_d_ and *V*_inter_ were calculated as 0.39 and 0.29 eV, respectively. Half the value of *V*_d_, i.e., 0.20 eV, closely matches the intradimer transfer integral *t*_d_, confirming that the conventional approximation *V*_d_ ≈ 2 *t*_d_ holds without significant contradiction. Furthermore, the value of *V*_inter_ is comparable to that of θ-type BEDT-TTF salts, which are known to exhibit charge ordering or charge fluctuations.^35,36^ This suggests the presence of charge inhomogeneity in κ-(BEDT-TTF)_2_[B_*R*/*S*_(salicylate)_2_]. The existence of charge fluctuations was further demonstrated through the analysis of intramolecular vibrations, which will be discussed in detail later.

One alternative evaluation method involves interpreting the MIR transition not as separate transitions but as an electronic transition from the lower to the upper Hubbard band. This approach is used when the two MIR transitions perpendicular to the stacking direction are so closely merged that curve fitting becomes impractical, as observed in the spectra of κ-(BEDT-TTF)_2_Cu_2_(CN)_3_.^26,28^ Under this interpretation, the MIR transition is treated as a single electronic transition, with the peak position representing the energy difference between the lower and upper Hubbard bands, which is proportional to *U*_d_ (=*V*_d_). Additionally, the half-width at half-maximum (HWHM) corresponds to the average bandwidth *W* of the two Hubbard bands, which is proportional to *t*′ and *t*. Using this approach, *U*_d_/*W* can be estimated from the maximum conductivity and HWHM.

Focusing on the *a*-polarized spectrum, as reported for κ-(BEDT-TTF)_2_Cu_2_(CN)_3_, the peak wavenumber of 3300 cm^–1^ and HWHM of 1450 cm^–1^ yield *U*_d_/*W* = 2.3. Based on this value alone, the system appears to exhibit the strongest electron correlation among previously reported materials.^28^ However, the *a*-polarized intensity is extremely weak, and the spectral shape differs from those of triangular or square lattice salts, suggesting that a direct comparison may not always be valid. In fact, estimation using the *b*-polarized spectrum yields *U*_d_/*W* = 1.3, based on wavenumbers of 2440 cm^–1^ and 1840 cm^–1^. This value is smaller than that of any previously reported material. The estimation of *U*_d_/*W* based on the previous study suggests that electronic correlations of κ-(BEDT-TTF)_2_[B_*R*/*S*_(salicylate)_2_] are strong in the *a* + *b* and *a* – *b* directions, while the correlation in the stacking *b*-direction is weak, which appears consistent with *t*′ > *t*. However, a large *t*′ relative to *V*_inter_ is inconsistent with the insulating behavior and the absence of a Drude component. This implies that the insulating behavior is not solely caused by electronic correlations but is also influenced by electron–lattice interactions along the stacking direction. This point will be further examined later in the analysis of intramolecular vibrations, particularly in light of the results indicating signs of tetramerization.

The broad range indicated by the dashed line in Figure 6a includes several peaks indicative of the Fano-type, suggesting the presence of a broad peak corresponding to the dashed line. These broad peaks are ν_3–3_ and ν_3–4_, as shown in Figure S5, and are characteristic of the IR reflectance spectra of most κ-type BEDT-TTF salts.^27,29^ In these intramolecular vibrations, the central C=C double bond of the two BEDT-TTF molecules in the dimer stretches in opposite phases.^37^ Due to the large electronic intramolecular vibration (e-mv) coupling constant of ν_3_ at 0.1 eV,^38^ ν_3–3_ and ν_3–4_ are much lower than the wavenumber 1470 cm^–1^ of ν_3_ for a single molecule corresponding to a +0.5 valence. To verify whether there are other intense anion-derived intramolecular vibrations in the same wavenumber region as ν_3–3_ and ν_3–4_, we compared the measured results with the results of the normal-mode analysis. Table S2 shows the results of the normal-mode analysis for [B(salicylate)_2_]^−^. Intramolecular vibrations marked with an asterisk in the table are also indicated by asterisks in the *b*-polarized conductivity spectra of Figure 6b, but their area intensities are negligible as they are three to 4 orders of magnitude smaller than those of ν_3–3_ and ν_3–4_. The peaks indicated by the dashed lines in Figure 6a are therefore assigned to ν_3–3_ and ν_3–4_. The estimated peak position of 1190 cm^–1^ for *b*-polarization is at a lower wavenumber than the estimated peak position of 1290 cm^–1^ for *a*-polarization. The peak intensity of *b*-polarization is greater than that of *a*-polarization. These behaviors are opposite to those of most κ-type BEDT-TTF salts.^25−30,37^ As shown in Figure S5, the phase relationship of the vibrations between the dimers ν_3–3_ and ν_3–4_ is different, so the amount of charge transfer induced between them by the e-mv interaction should be different. Therefore, the fact that the polarization dependence is opposite to that of most κ-type BEDT-TTF salts is consistent with a larger interdimer transfer integral in the stacking direction.

**Figure 7:**
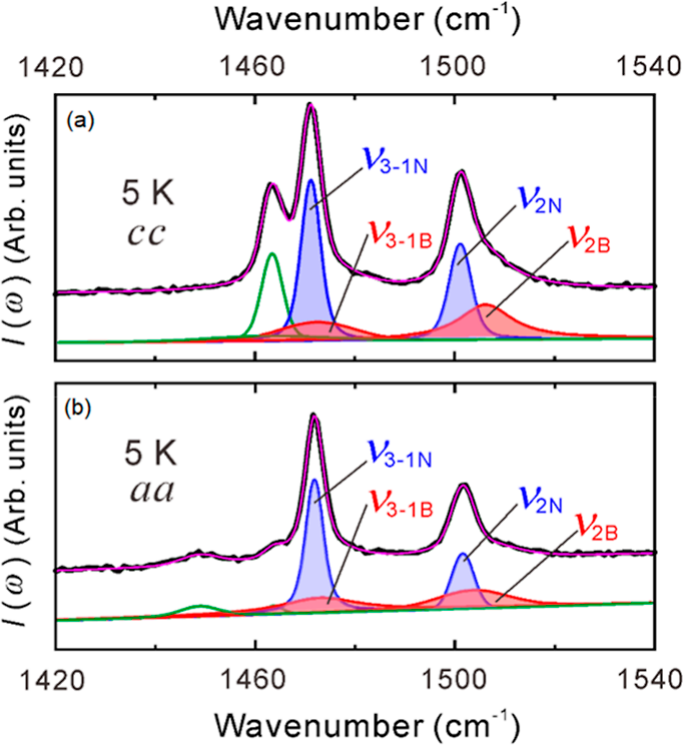
Results of the curve fittings for the (a) *cc*- and (b) *aa*-polarized Raman spectra. In both (a) and (b), the top overlapping black and pink curves represent the experimental data and the summation of the separated components, respectively. The bottom curves denote the separated components. The top and bottom curves are offset. The mode ν_2_ consists of broad and narrow components, ν_2B_ and ν_2N_, respectively. The mode ν_3–1_ consists of broad and narrow components, ν_3–1B_ and ν_3–1N_.

Figure S6 presents the polarization dependence of the Raman spectra measured at 5 K. In the range from 1100 to 1600 cm^–1^, there are no strong intramolecular vibrations originating from [B(salicylate)_2_]^–^. The polarization dependence is consistent with the space group *P*2_1_. As described in the Supporting Information, a comparison of the wavenumber of ν_3–2_ with that in κ-(BEDT-TTF)_2_Cu[N(CN)_2_]Br supports the conclusion that the value of *t* for the title compound is smaller.^37^ The ν_3–1_ mode is the only mode in which all BEDT-TTF molecules in the unit cell vibrate in phase.^37^Figure 7 shows an expanded view of the wavenumber region where ν_3–1_ and ν_2_ are observed, based on two of the polarized spectra presented in Figure S6. Ideally, ν_3–1_ should appear as a single sharp peak if every unit cell is homogeneous and all molecules in the unit cell vibrate in-phase. However, instead of a single sharp peak, a broad peak also seems to be present. Similar behavior is observed in the wavenumber range where ν_2_ is observed.

Curve fitting was performed to separate the multiple components of the *cc*- and *aa*-polarized spectra. None of the peaks could be fitted with the Lorentz function alone, suggesting that the 2D conduction layer is an inhomogeneous system. They could, however, be fitted with either the Voigt function or the Gaussian function. The results fitted by the Voigt function are shown in Figure 7. ν_3–1_ was found to consist of a broad line width component, ν_3–1B_, and a narrow line width component, ν_3–1N_. Similarly, ν_2_ was found to consist of a broad line width component, ν_2B_, and a narrow line width component, ν_2N_. Since ν_3–1_ is entirely in-phase, it does not directly reflect the influence of the transfer integral between dimers as ν_3–2_ to ν_3–4_ do. Therefore, the phenomenon of multiple peaks must be influenced by other factors. The ν_2_ wavenumber is proportional to the charge of the molecules.^39^ Two peaks, ν_2N_ and ν_2B_, were observed; however, these peaks are not considered indicative of charge separation. This is because the peak position of ν_2N_ is precisely at the +0.5 charge position. Interpreting the splitting into ν_2N_ and ν_2B_ as resulting from charge inhomogeneity would contradict stoichiometric principles. A similar phenomenon has been observed in salts with triangular lattices of *monoclinic*- and *triclinic*-EtMe_3_P[Pd(dmit)_2_]_2_ where the high-wavenumber peak, analogous to ν_2B_, is explained as a mode in which two dimers within a tetramer stretch in phase.^40,41^ In this mode, the electron–molecular vibration interaction between dimers is enhanced, leading to a higher wavenumber. In contrast, ν_2N_ remains at the wavenumber corresponding to a +0.5 charge and represents a dimer that does not form a tetramer.

The line widths of ν_2N_ and ν_2B_ are different, so the presence of a broad peak, ν_2B_, indicates a large distribution in the charge of the BEDT-TTF molecules in the crystal. Conversely, the presence of a peak with a narrow line width, ν_2N_, indicates a small distribution in the charge of the BEDT-TTF molecule. The coexistence of ν_2B_ and ν_2N_ implies that regions with large and small charge distributions of the BEDT-TTF molecule coexist in the crystal. The wavenumber of ν_2_ is simply proportional by 120 cm^–1^ between BEDT-TTF^0^ and BEDT-TTF^+^.^39^ The line widths of ν_2B_ and ν_2N_ are 16.0 cm^–1^ and 5.0 cm^–1^, resulting in standard deviations of the charge values of 0.06 and 0.02, respectively. It is suggested that ν_3–1_ also consists of ν_3–1B_ and ν_3–1N_ for the same reason. The coexistence of regions with different charge distributions has not been observed before in triangular lattices with low dimensionality that become nonmagnetic or charge-ordered at low temperatures, such as κ-(BEDT-TTF)_2_B(CN)_4_ and κ-(BEDT-TTF)_2_Hg(SCN)_2_Cl.^31,42^ Instead, this phenomenon is observed in EtMe_3_Sb[Pd(dmit)_2_]_2_, κ-(BEDT-TTF)_2_Cu_2_(CN)_3_ and κ-(BEDT-TTF)_2_Ag_2_(CN)_3_,^43−45^ which retains spin liquid behavior at low temperatures, as seen in its magnetic susceptibility at low temperatures.^46−49^ This behavior is extremely interesting as it provides a clue for interpreting experimental results showing that magnetic susceptibility decreases at low temperatures but does not reach zero. The coexistence ν_2N_ and ν_2B_, attributed to the coexistence of dimer and tetramer structures, respectively, is also observed in other triangular lattice systems, such as EtMe_3_As[Pd(dmit)_2_]_2_ and κ-(BEDT-TTF)_2_Cu[N(CN)_2_]I.^50,51^

A stronger frustration in the magnetic susceptibility was suggested was observed for κ-(BEDT-TTF)_2_[B_*R*/*S*_(salicylate)_2_] than for κ-(BEDT-TTF)_2_B(CN)_4_, even though the dimensionality of κ-(BEDT-TTF)_2_[B_*R*/*S*_(salicylate)_2_] is lower than that of κ-(BEDT-TTF)_2_B(CN)_4_.^31,52^ The [B(salicylate)_2_]^–^ is incorporated into the crystal in a mixture of *S*- and *R*-enantiomers as a result of the use of chiral molecules for the anions. The introduction of the disordered state by the chiral molecule prevents the two-dimensional layer from being homogeneous and is thought to result in an electronic state similar to that of a QSL. A material that behaves like a spin liquid by introducing a disordered state is EtMe_3_Sb[Pd(dmit)_2_]_2_, but unlike κ-type BEDT-TTF salts, it becomes nonmagnetic or antiferromagnetic even if the 2D layer deviates from the regular triangular lattice by only a few percent,^53,54^ so a spin liquid cannot be realized unless the disordered state is introduced under conditions very close to those of an equilateral triangular lattice. A significant finding of this study is the realization of electronic states resembling QSLs, achieved even in structures that markedly deviate from an equilateral triangular lattice, through the introduction of enantiomers.

### Heat Capacity

There are a handful of QSL materials that have previously been reported in organic molecular dimer Mott insulators, such as κ-(BEDT-TTF)_2_Cu_2_(CN)_3_,^13^ κ-(BEDT-TTF)_2_Ag_2_(CN)_3_,^14^ EtMe_3_Sb[Pd(dmit)_2_]_2_,^15^ κ-H_3_(Cat-EDT-TTF)_2_.^16^ In these molecular solids the heat capacity behavior shows gapless excitation character,^23,55,56^ and the Wilson Ratio obtained from the coefficient γ of the *T*-linear term and χ_0_ in the magnetic susceptibility measurement are close to 1. From a thermodynamic point of view Fermi liquid like character has been reported. The absolute values of γ are proportional to 1/*J*, therefore a system with weaker *J* values demonstrates a larger γ value. (Et-4IT)[Ni(mnt)_2_]_2_ is a recent QSL candidate with weaker interactions, having a value of γ of about 10^2^ mJK^–2^ mol^–1^.^57^ This value cannot be explained without consideration of the origin of the magnetism. The gapless behavior of the QSL state based on dimer Mott insulators is intrinsic. The detection of gapped behavior has also been suggested by other measurements,^58−60^ however, around 3–6 K, a broad thermal anomaly is also observed in heat capacity measurements.^55^ Sometimes this broad thermal anomaly is referred to as the 6 K anomaly in QSL systems. This anomaly has also been reported in EtMe_3_Sb[Pd(dmit)_2_]_2_.^23^ Therefore, there is a possibility of the coexistence of gapless excitation and small gapped excitation in organic QSL systems. In other words, both gapped excitation with T-linear coefficient and small gapped behavior with a broad thermal anomaly can be considered as the thermodynamic character of the QSL system. It was recently reported that the spin gap is intrinsic to κ-(BEDT-TTF)_2_Cu_2_(CN)_3_, but that the amount of extrinsic impurities and disorder may cause variation from one single crystal sample to another.^61^ The origin of the 6K anomaly is also debated, with one possibility being a type of BKT transition (Berezinskii–Kosterlitz–Thouless transition).^62,63^ The gapless QSL state is achieved through a very delicate balance, and there is a possibility that a gap structure can be easily realized. It is therefore interesting to see what characteristics are detected owing to the anisotropic triangular structure and chiral structure in this new material, κ-(BEDT-TTF)_2_[B_*R*/*S*_(salicylate)_2_].

**Figure 8:**
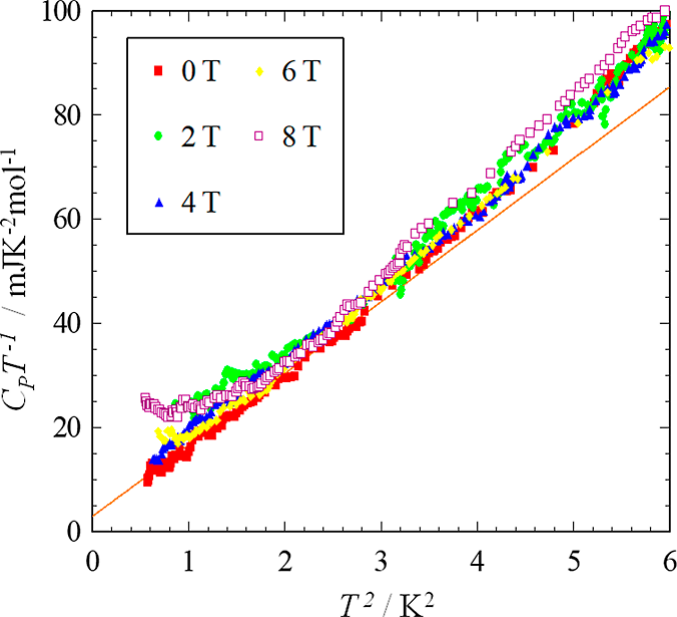
C_p_T^–1^ vs T^2^ plot for κ-(BEDT-TTF)_2_[B_*R/S*_(salicylate)_2_] under applied magnetic fields.

In Figure 8, the temperature dependence of the heat capacity of κ-(BEDT-TTF)_2_[B_*R*/*S*_(salicylate)_2_] was plotted using C_p_T^–1^ vs T^2^ plot below 6 K^2^ (2.45 K). The red line indicates the fitting result by using the formula C_p_T^–1^ = AT^–3^ + γ + βT^2^ with the data below 3 K^2^ at 0 T. In this formula the AT^–3^, γ and βT^2^ terms are the nuclear Schottky tail heat capacity, the T-linear contribution usually observed in metallic state or gapless spin liquid state, and the lattice contribution at low temperature region, respectively. We have obtained the parameters as A = 0.58 mJKmol^–1^, γ = 1.33 mJK^–2^ mol^–1^, and β = 14.2 mJK^–4^ mol^–1^. We also plot the data under applied magnetic fields. The upturn structures observed under 6 and 8 T were considered as nuclear spin Schottky tail heat capacity. A small and broad upturn structure appeared under 2 T and the broad small hump structure under 4 T indicating the existence of a small amount of a paramagnetic-like component. Impurities or lattice defects sometimes produce a paramagnetic-like spin component in the sample. The total amount of the paramagnetic spins is less than 0.5% of Rln2. Probably the reason for the small finite value of γ under 0 T is due to the paramagnetic-like spin effect for the heat capacity. When this kind of spin is produced in the conductive layer, the spins are affected by surrounding spins. The spins cannot be perfectly isolated and have small interactions. The degree of the interactions depends on the situation of each spin, therefore the temperature dependence due to paramagnetic spins sometimes demonstrates T-linear-like behavior with a small γ value usually less than 5 mJK^–2^ mol^–1^ at low temperature. This behavior also shows clear field dependence in κ-(BEDT-TTF)_2_[B_*R*/*S*_(salicylate)_2_] therefore this effect can be distinguished easily from an intrinsic gapless character. From these considerations, we conclude that the γ value of κ-(BEDT-TTF)_2_[B_*R*/*S*_(salicylate)_2_] is not intrinsic and negligible. Therefore, we have to conclude that the system is not a gapless spin liquid.

**Figure 9:**
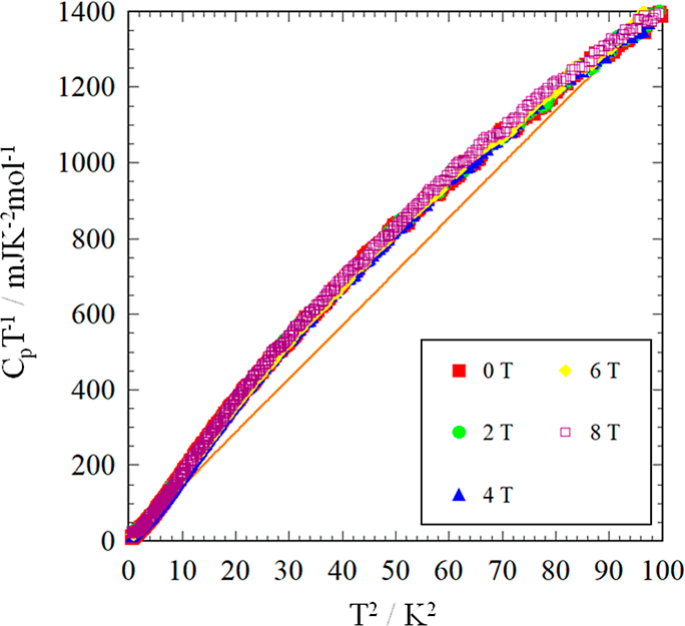
C_p_T^–1^ vs T^2^ plot for κ-(BEDT-TTF)_2_[B_*R/S*_(salicylate)_2_] under applied magnetic fields with the fitting line indicated by the orange line the same as for Figure 9.

**Figure 10:**
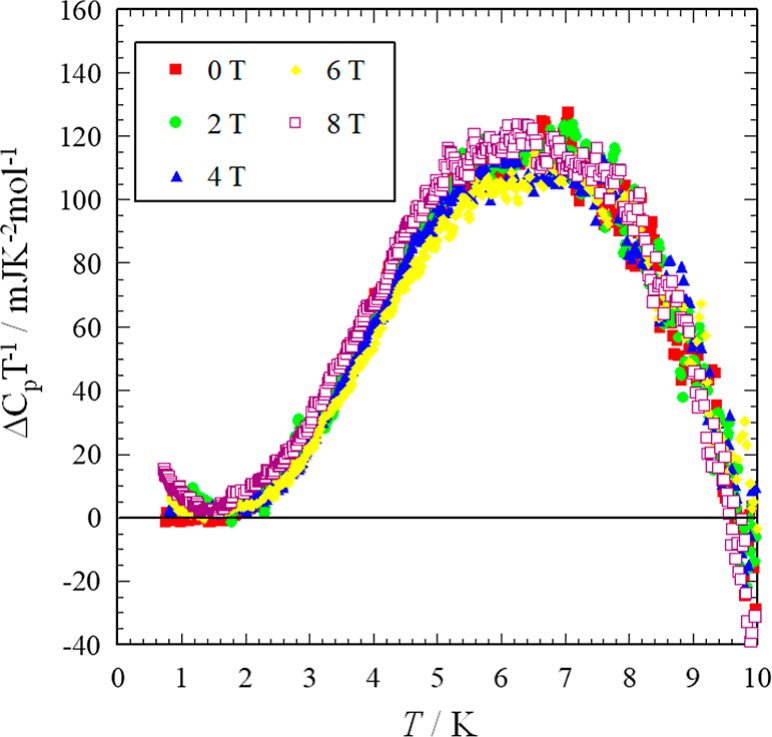
C_p_T^–1^ vs T plot for κ-(BEDT-TTF)_2_[B_*R/S*_(salicylate)_2_] under applied magnetic fields.

On the other hand, the C_p_T^–1^ values are remarkably larger than the fitting line above 1.73 K under all magnetic fields. In Figure 9, the heat capacity data under applied magnetic field were plotted in C_p_T^–1^ versus T^2^ plots with the fitting line indicated by the orange line, the same as Figure 8. The C_p_T^–1^ values were remarkably larger than the fitting line around 7 K. This behavior indicates the existence of the broad thermal anomaly at higher temperature regions similar to κ-(BEDT-TTF)_2_Cu_2_(CN)_3_ and EtMe_3_Sb[Pd(dmit)_2_]_2_.^23,55^ In order to estimate the detail of the thermal anomaly we have estimated the difference between the data and the fitting line by ΔC_p_T^–1^, which was estimated by ΔC_p_T^–1^ = C_p_T^–1^ – (AT^–3^ + γ + βT^2^). In Figure 10, we have plotted the ΔC_p_T^–1^ of κ-(BEDT-TTF)_2_[B_*R*/*S*_(salicylate)_2_]. There is a broad peak structure and the peak top temperature is 7.0 K. In this plot and analysis, we have to be careful of the possibility of underestimation - in the case of a typical dimer (BEDT-TTF)_2_X system which does not show this kind of behavior.^55^ The ΔC_p_T^–1^ indicates almost 0 or a negative value, which is similar to the behavior of ΔCpT^–1^ for X[Pd(dmit)_2_]_2_ systems. Antiferromagnetically ordered systems and charge-ordered systems show negative values,^23^ with the negative values appearing above 6 K. Therefore, when the peak top temperature is higher than 6 K, we cannot estimate the correct thermal anomaly. The peak top temperature can be a higher value with the correct estimation such as using the heat capacity of an ordinary Mott insulating system with a similar anion and crystal structure. Moreover, the peak height of the anomaly of κ-(BEDT-TTF)_2_[B_*R*/*S*_(salicylate)_2_] is 2–3 times larger than that observed for κ-(BEDT-TTF)_2_Cu_2_(CN)_3_ and EtMe_3_Sb[Pd(dmit)_2_]_2_.^23,55^ In general, a larger peak structure and a higher peak top temperature indicates a larger gap formation. Therefore, the conclusion of gapped behavior for κ-(BEDT-TTF)_2_[B_*R*/*S*_(salicylate)_2_] with a larger broad thermal anomaly is reasonable.

In contrast to this large thermal anomaly, another QSL system (Et-4IT)[Ni(mnt)_2_]_2_ does not show any hump structure in the heat capacity.^57^ This system shows a remarkably large γ value with gapless spin liquid behavior due to the weak interaction. We can speculate upon the close relationship between the degree of the gapless excitation and the thermal anomaly in terms of entropy. In other words, the total entropy composed of gapless excitation and thermal anomaly may be almost kept in frustrated systems. In addition, we have to pay attention to the absence of the thermal anomaly in other Mott insulating systems with less frustrated systems. Therefore, when the system has strong frustration which prohibits formation of typical magnetic ordering, the remaining entropy at low temperature, mainly below 10 K, becomes significantly large. In this case, we speculate that the possible origin of the thermal anomaly is a kind of BKT-like transition in frustrated systems. When we assume that the degree of the BKT transition affects the γ values, moreover to determine the excitation structure. When the BKT transition cannot form clear ordering due to strong enough frustration, the system can be a gapless QSL state. In this case, the thermal anomaly will be broad and relatively small as for EtMe_3_Sb[Pd(dmit)_2_]_2_. When the frustration effects becomes weaker due to a reason such as minor crystal structure changes, this transition behavior becomes sharp and a gapped system is constructed. This may be the case for the gapped behavior in κ-(BEDT-TTF)_2_Cu_2_(CN)_3_ based on this assumption,^58^ and also thermodynamic behavior may be sharpened in this case. The behavior of κ-(BEDT-TTF)_2_[B_*R*/*S*_(salicylate)_2_] resembles this case, and it is reasonable to observe the larger broad thermal anomaly with gapped behavior without a clear transition detected by magnetic susceptibility measurements. Of course this is speculation, however this discussion gives us an important possibility for κ-(BEDT-TTF)_2_[B_*R*/*S*_(salicylate)_2_]. The observation of the broad thermal anomaly indicates that κ-(BEDT-TTF)_2_[B_*R*/*S*_(salicylate)_2_] is really close to a gapless QSL state. Only a small structural change can lead to this state considering the gapped behavior in κ-(BEDT-TTF)_2_Cu_2_(CN)_3_. An intentional phase control around a gapless QSL and gapped spin QSL system will play an important role in answering many questions about QSLs.

**Figure 11:**
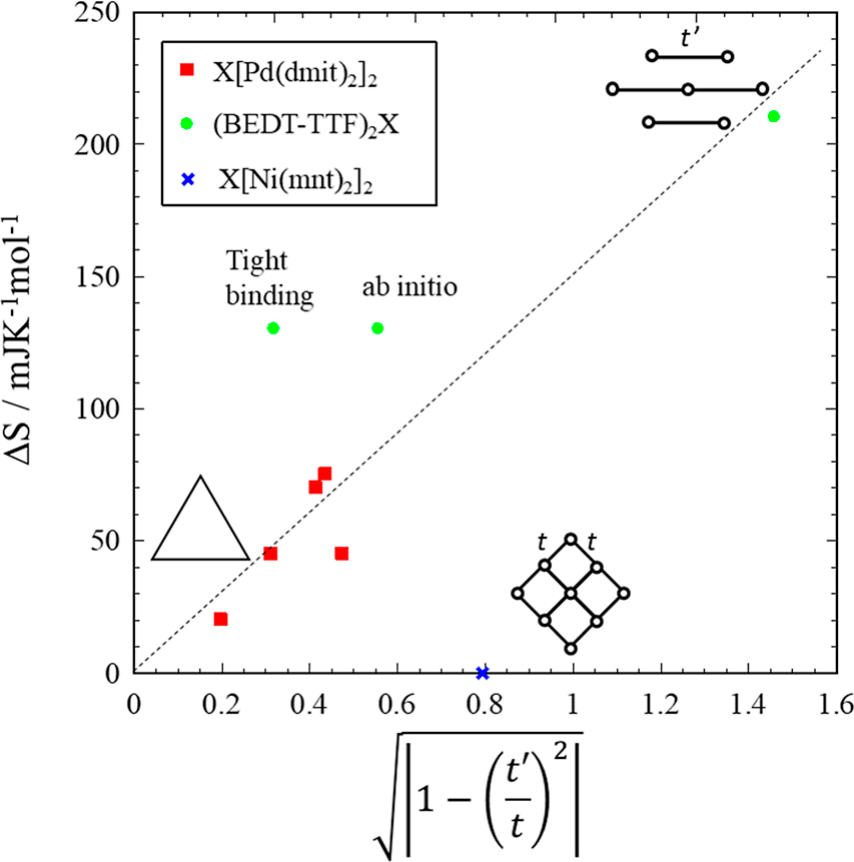
Relationship of the entropy change, Δ*S*, due to the anomaly and the anisotropy of the triangle structure based on the magnetic interactions .

We have also investigated the relationship between the anomaly and the electronic structure. In Figure 11, the entropy change Δ*S* due to the anomaly and the anisotropy of the triangle structure  (square lattice side) and  (1D chain side or Valence Bond Solid side). In other words, this parameter is the magnitude of the difference of the magnetic interaction between each side of the triangles (*J*′–*J*). The value of parameter 0 corresponds to a regular triangular lattice, around this point gapless spin liquids have been observed. Therefore, we used the parameter of  to scale the magnitude of the anomaly. The red squares on the square lattice side correspond to (EtMe_3_Sb)_1–*x*_(Et_2_Me_2_Sb)_*x*_[Pd(dmit)_2_]_2_ system and the (EtMe_3_Sb)_1–*y*_(Me_4_Sb)_*y*_[Pd(dmit)_2_]_2_ system, these are the solid solution crystals composed of the spin liquid system and the charge ordered state and the solid solution crystals composed of the spin liquid system and the antiferromagnetic ordered system, respectively. The green circles are κ-(BEDT-TTF)_2_Cu_2_(CN)_3_ and the present system. Δ*S* values were calculated from ΔC_p_T^–1^ of each system, these are estimated by the same method as that used to obtain Figure 10. For κ-(BEDT-TTF)_2_Cu_2_(CN)_3_, there are two types of the *t*′/*t* value based on tight binding model and ab initio calculation, respectively. According to present discussion, the ab initio model with *t*′/*t* = 0.83 seems to be reasonable. In Figure 11, the value of Δ*S* increased as the absolute value of the  increased. In other words, when the system is far from an equilateral triangle larger values of Δ*S* are observed. A similar trend exists for the anomaly peak temperature. The larger absolute value gives the higher peak top temperature of the anomalies. Therefore, the anomaly may be related to the anisotropy of the regular triangular lattice. On the other hand, Δ*S* of (Et4-BrT)[Ni(mnt)_2_]_2_, which is located closest to the square lattice side, was zero. There is no broad thermal anomaly in the heat capacity. Probably, the reason for this is thought to be related to the magnitude of the antiferromagnetic interaction *J*. For other spin liquid systems, the interactions are approximately *J*/k_B_ = –200–300 K, this value is almost 10 times stronger than that of (Et4-BrT)[Ni(mnt)_2_]_2_. In addition to the triangular lattice property, parameters such as antiferromagnetic interaction are considered to be related to the anomaly.

As discussed above, there may be a relationship between the magnitude of the anomaly and the gapless excitation behavior at low temperatures. In this case, it can be understood that there is a tendency for gaps to open more easily when the lattice deviates from the regular triangular lattice. From this viewpoint, it is interesting to compare (Et4-BrT)[Ni(mnt)_2_]_2_ and the present system. Both materials deviate sufficiently from the regular triangular lattice, usually it is little bit difficult to imagine spin liquid realization. However, (Et4-BrT)[Ni(mnt)_2_]_2_ has clear spin liquid state behavior with gapless excitations. On the other hand, although there is no clear evidence of a gapless spin liquid, the thermal anomaly, which is a kind of spin-liquid feature and is not observed in a typical antiferromagnetic system, has also been observed in the present system.

The common feature of these systems is that the anion has remarkable anisotropy, which affects the crystal structure of the conductive layer. In (Et4-BrT)[Ni(mnt)_2_]_2_, a bilayer system with clearly different crystal structure and electronic structure has been realized. This is thought to inhibit long-range order formation. On the other hand, in the present system, dimers are formed by crystallographically distinct molecules in the conductive layer due to chirality. Although this is speculation, it can be considered that a bilayer system exhibits a larger long-range order inhibition effect, and that the chirality-based system has not been able to completely realize a gapless spin liquid but realize the broad thermal anomaly as a part of the spin liquid character. Therefore, again it can be considered that the present system is very close to a gapless spin liquid. There is the possibility of gapless spin liquid behavior by introducing greater anisotropy.

In conclusion, this new system, κ-(BEDT-TTF)_2_[B_*R*/*S*_(salicylate)_2_], offers the potential to be tuned through chemical pressure by adapting the salicylate ligand with atom-by-atom changes to tune the structure and tailor of a family of organic molecular materials which lie in and around the QSL region of the phase diagram for these salts. This offers the prospect for an experimental playground to deepen understanding in the QSL state.

## Experimental Section

### Synthesis of κ-(BEDT-TTF)_2_[B_*R/S*_(salicylate)_2_]

Crystals were grown on platinum electrodes by electrocrystallization in 40 mL H-shaped electrochemical cells. Platinum electrodes were cleaned by applying a voltage across the electrodes in 1 M H_2_SO_4_ in each direction to produce H_2_ and O_2_ at the electrodes, then washed with distilled water and thoroughly dried.

TEA [B_*R/S*_(salicylate)_2_] (250 mg) was dissolved in chlorobenzene (30 mL) with stirring overnight before filtering into an H-cell containing BEDT-TTF (10 mg) in the anode compartment. H-cells were placed in a dark box on a vibration-free bench at a constant current of 0.5 μA and after 21 days a large number of black hexagonal crystals of κ-(BEDT-TTF)_2_[B_*R/S*_(salicylate)_2_] were harvested from the anode.

#### Crystal Data for κ-(BEDT-TTF)_2_B_*R/S*_(salicylate)_2_

C_34_H_24_O_6_S_16_B_1_, M = 1052.30, black hexagon, *a* = 11.0971(3), *b* = 8.3292(2), *c* = 22.3188(5) Å, α = 90, β = 101.740(2), γ = 90°, *U* = 2019.78(9) Å^3^, *T* = 150 K, space group *P2*_1_, Z = 2, μ = 0.903 mm^–1^, reflections collected = 11,415, independent reflections = 7913, *R*1 = 0.0736, w*R*2 = 0.1133 [*F*^2^ > 2σ(*F*^2^)], *R*1 = 0.1489, w*R*2 = 0.1698 (all data). CCDC 2339767.

### Physical Measurements

X-ray data were collected on a Rigaku Oxford Diffraction Xcalibur System equipped with a Sapphire detector at room temperature using MoKα radiation (λ = 0.71073 Å) with CrysAlisPRO software.^64^

Four-probe DC transport measurements were made on crystals using a HUSO HECS 994C multichannel conductometer. Gold wires (15 μm diameter) were attached to the *ab* face of the crystal and resistivity was measured along the *b* direction. The attached wires were connected to an integrated circuit plug with carbon conductive cement.

Magnetic susceptibility measurements were performed with a Quantum Design MPSM2 SQUID magnetometer using randomly orientated polycrystalline material encased in aluminum foil.

Polarized IR reflectance spectra were acquired using a JASCO FT/IR-8X spectrometer and an IRT-5200 infrared microscope. The IR spectra were subsequently transformed into conductivity spectra using the Kramers–Kronig transformation. Raman spectra were recorded using a RENISHAW inVia Reflex system in a backscattering configuration. Single crystals for the Raman spectra were cooled to 5 K using a helium-flow cryostat at a cooling rate of 1 K/min.

The heat capacity measurements have been performed by a homemade relaxation calorimeter. The calorimeter was composed of two RuOx chips, working as the thermometer and the heater, respectively. Both RuOx chips were made by KOA corporation. The resistance of the thermometer chip and the resistance of the heater at room temperature was 10 kΩ and 1 kΩ, respectively. The heater also worked as the sample stage. We measured the heat capacity of κ-(BEDT-TTF)_2_[B_*R*/*S*_(salicylate)_2_] using a single crystal weighing 0.7224 mg. The crystal was attached to the sample stage with Apiezon N grease. The calorimeter was attached to the top-loading insert probe and the probe was sealed and inserted into a VTI system with superconducting magnet. The heat capacity was then measured in the temperature range 0.7 to 10 K under magnetic fields of up to 10 T.

## Supporting Information Available

Table of S···S contacts, ORTEP image of the asymmetric unit, image of the S and R enantiomers of B(salicylate)_2_, images of the crystal structure viewed down the *b* and the *c* axes, image of the vibrational motions of BEDT-TTF, table of calculated vibrational wavenumbers of [B(salicylate)_2_]^−^, and polarized Raman spectra for κ-(BEDT-TTF)_2_[B_*R/S*_(salicylate)_2_] (PDF)
