# Correction to “2D Quantum Spin-Liquid Candidate Including a Chiral Anion: κ‑(BEDT-TTF)_2_[B_*R*/*S*_(salicylate)_2_]”

**DOI:** 10.1021/jacs.6c07716

**Published:** 2026-04-28

**Authors:** Toby J. Blundell, Kathryn Sneade, Joseph O. Ogar, Satoshi Yamashita, Hiroki Akutsu, Yasuhiro Nakazawa, Takashi Yamamoto, Lee Martin

There is an addition to the Acknowledgment: H.A. and L.M. would like to thank JSPS for financial support (JSPS Core-to-Core Program, grant number: JPJSCCA20240001).

