# Supplementary material for: 2D Quantum Spin-Liquid Candidate Including a Chiral Anion: κ-(BEDT-TTF)2[BR/S(salicylate)2]
Source: J Am Chem Soc. 2025 Feb 6;147(7):5658–68. doi: 10.1021/jacs.4c12386 (PMC11848823; doi:10.1021/jacs.4c12386)
Supplement: Supplementary file 1 — ja4c12386_si_001.pdf [file ja4c12386_si_001.pdf]

2D Quantum Spin-Liquid Candidate including a chiral anion - κ-(BEDT-TTF)$_2$[B$_{R/S}$(salicylate)$_2$]

Toby J. Blundell,[a] Kathryn Sneade,[a] Joseph O. Ogar,[a] Satoshi Yamashita,[b] Hiroki Akutsu,[b] Yasuhiro Nakazawa,[b] Takashi Yamamoto[c,d] and Lee Martin[a*]

[a] *School of Science and Technology, Nottingham Trent University, Clifton Lane, Clifton, Nottingham, NG11 8NS, UK*
[b]*Department of Chemistry, Graduate School of Science, Osaka University, 1-1 Machikaneyama-cho, Toyonaka, Osaka 560-0043, Japan*
[c]*Graduate School of Science and Engineering, Ehime University, Matsuyama, 790-8577, Japan*
[d]*Geodynamics Research Center, Ehime University, Matsuyama, 790-8577, Japan*
Corresponding Author: Lee Martin ORCID ID 0000-0002-5330-5700, E-mail lee.martin@ntu.ac.uk

Supporting Information

Table S1 S…S contacts shorter than the van der Waals distance for κ-(BEDT-TTF)$_2$[B$_{R/S}$(salicylate)$_2$].

S…S	Å at 150K
S11…S16	3.532(5)
S1…S12	3.368(4)
S1…S10	3.396(5)
S5…S16	3.532(5)
S7…S16	3.431(5)
S8…S13	3.534(4)
S8…S15	3.446(5)
S4…S9	3.386(4)
S2…S9	3.418(5)
S1…S15	3.587(5)
S7…S9	3.580(5)
S3…S8	3.569(5)

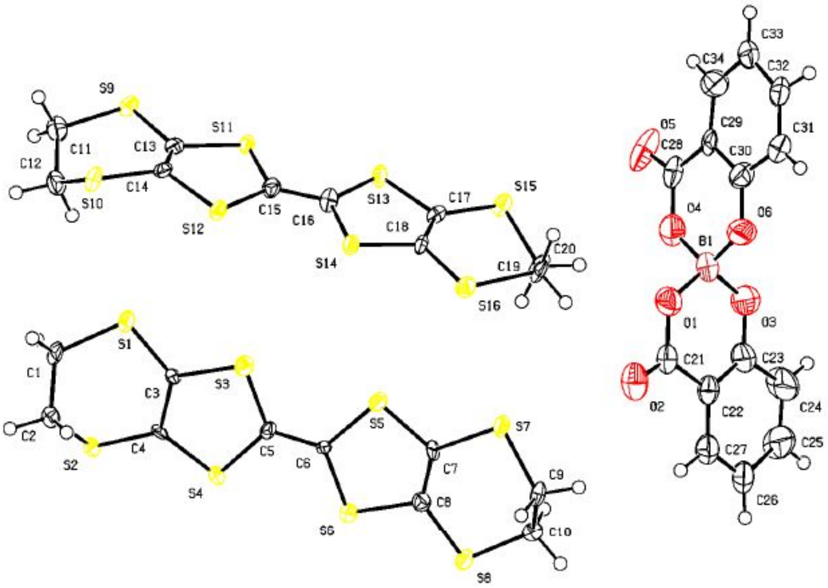

Fig. S1 Asymmetric unit of κ-(BEDT-TTF)$_2$[B$_{R/S}$(salicylate)$_2$]. Thermal ellipsoids set at 50% probability.

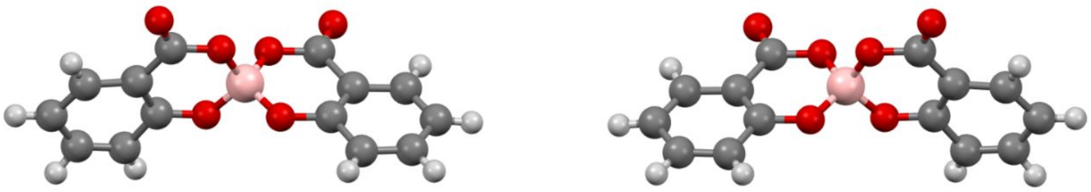

Fig. S2 B_S(salicylate)$_2$ (left) and B_R(salicylate)$_2$ (right).

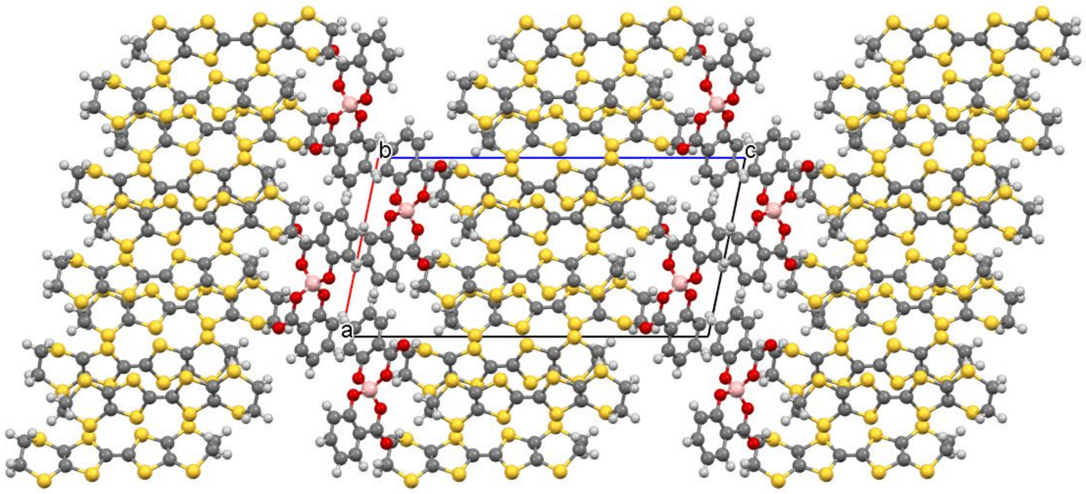

Fig. S3 Layered structure of κ-(BEDT-TTF)$_2$[$B_{R/S}$(salicylate)$_2$] viewed down the *b* axis.

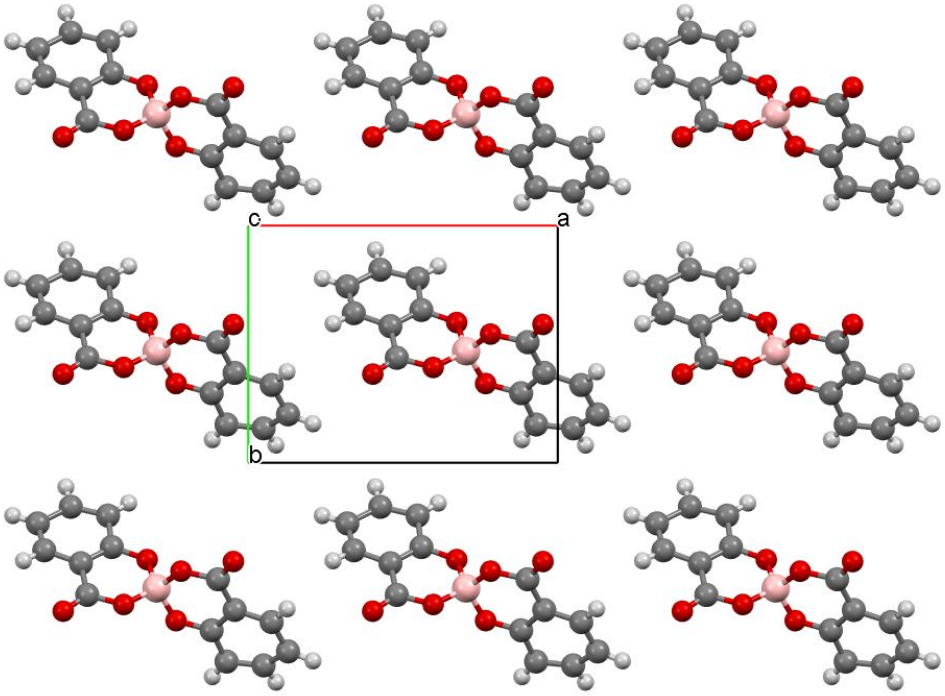

Fig. S4 Anion layer of κ-(BEDT-TTF)$_2$[$B_{R/S}$(salicylate)$_2$] viewed down the *c* axis.

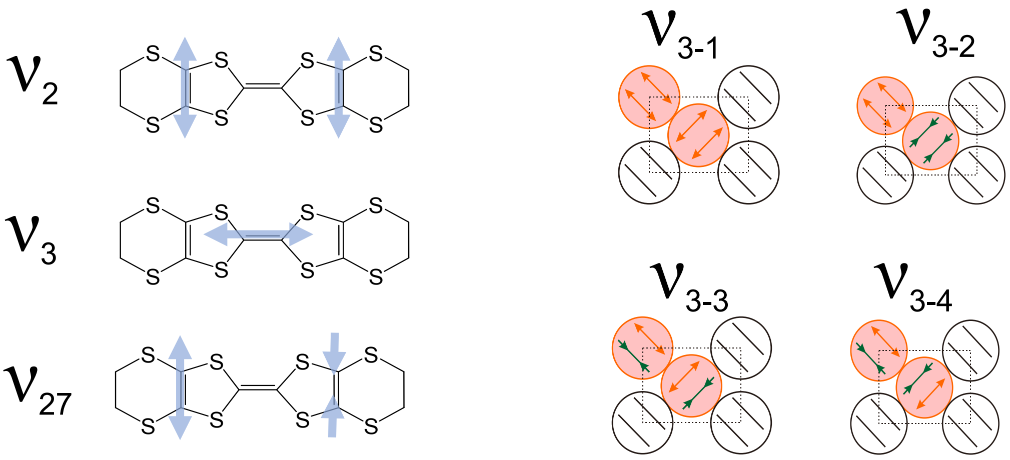

Fig. S5 Vibrational motions of ν2, ν3, and ν27, along with the four vibrational modes originating from the ν3 mode produced by the four BEDT-TTF molecules in the repeating unit of the conducting layer. Circles denote dimers, and arrows within the filled circles indicate the phases of the C=C stretching vibrations.

Table S2 Calculated vibrational wavenumbers of $[B(salicylate)_2]^-$ between 1000 cm^{-1} and 1700 cm^{-1}, relative IR intensity, and relative Raman activity. These values were obtained using Gaussian 06 programs with DFT-B3LYP theory and the 6-31G+(d,p) basis set.[S1]The scaling factor 0.964 was applied. Asterisks in the middle column denote the vibrational modes observed in the *b*-polarized spectra of Fig. 6(b).

Wavenumber (cm^{-1})	Relative IR intensity	Relative Raman activity
1012	5	61
1031	1036*	3
1112	382	2
1220	274	3
1253	51	88
1298	1168*	1
1313	10	76
1443	264	1
1457	343	4
1556	14	36
1592	327*	46
1596	21	38
1672	602*	41
1688	421*	59

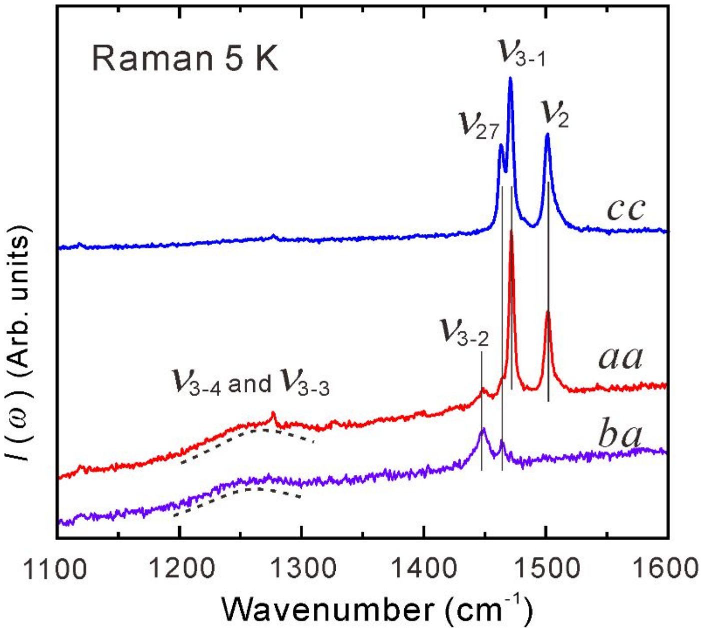

Fig. S6 Polarized Raman spectra for κ-(BEDT-TTF)$_2$[B$_{R/S}$(salicylate)$_2$]. The vibrational motions of ν_2, $\nu_{3\text{-}1}$, ν_{27}, and $\nu_{3\text{-}2}$ are depicted in Fig. S5. Dashed lines in the *aa*- and *ba*-polarized spectra indicate the $\nu_{3\text{-}4}$ and $\nu_{3\text{-}3}$ modes, respectively, as shown in Fig. S5

Figure S6 shows the polarized Raman spectra measured at 5 K. The first letter of *cc*, *aa*, and *ba* denotes the direction of polarization of the incident light, while the last letter denotes the direction of polarization of the scattered light. Comparing the Raman spectra with the results of the normal mode analysis of [B(salicylate)$_2$]$^-$, shown in Table S2, reveals that there are no intense [B(salicylate)$_2$]$^-$ peaks, and their intensity is negligible compared to the peaks associated with the BEDT-TTF vibrations. Furthermore, according to the normal mode analysis results, there are no conspicuous intensity peaks in the range of 1313 cm^{-1} to 1556 cm^{-1}. Therefore, the Raman peaks observed around this wavenumber range can be attributed to the BEDT-TTF molecule.

In the *aa*- and *ba*-polarized spectra, those which are polarization spectra within the conduction plane, $\nu_{3\text{-}3}$ and $\nu_{3\text{-}4}$ are observed, although their relative intensity is weaker than other peaks. They are originally strongly observed in the IR reflectance spectrum, but since the crystals of κ-(BEDT-TTF)$_2$[B$_{R/S}$(salicylate)$_2$] belong to the space group $P2_1$ with no inversion symmetry centre, it is expected that they are observed in the Raman spectrum. The mode ν_{27}, theoretically a strong IR mode, is also observed in the Raman spectrum with non-negligible strength, which is consistent with the $P2_1$ space group.

The mode $\nu_{3\text{-}2}$ is observed in the Raman spectrum because the BEDT-TTF molecules in the dimer vibrate in-phase. The wavenumber of this mode is 1449 cm^{-1}, lower than the wavenumber of 1470 cm^{-1} for ν_3 in the +0.5-valence single BEDT-TTF molecule. This is due to inter-dimer charge transfer in the *a*+*b* and *a*-*b* directions caused by the opposite phase between the dimers. The wavenumber of 1449

cm^{-1} for this mode is noticeably higher than the wavenumber of 1420 cm^{-1} for representative κ-type BEDT-TTF salts, such as κ-(BEDT-TTF)$_2$Cu[N(CN)$_2$]Br.[S2] Due to the electron-molecular vibration (e-mv) interaction, this frequency decreases to below a wavenumber of +0.5, as the two dimers vibrate in opposite phases. The magnitude of this frequency shift is known to be proportional to the transfer integral between the dimers.[S3] This phenomenon is consistent with the calculated *t*, indicating that the averaged *t* for κ-(BEDT-TTF)$_2$[B$_{R/S}$(salicylate)$_2$] is 30% smaller than that of κ-(BEDT-TTF)$_2$Cu[N(CN)$_2$]Br.[S4]

The mode $\nu_{3\text{-}1}$, like $\nu_{3\text{-}2}$, is a vibrational mode where the BEDT-TTF molecules in the dimer vibrate in-phase; however, unlike $\nu_{3\text{-}2}$, it is the only mode where all BEDT-TTF molecules in the unit cell vibrate in-phase. The mode $\nu_{3\text{-}1}$ is not observed in the *ba*-polarized spectrum because the space group $P2_1$ is consistent with it being in point group C_2. Additionally, $\nu_{3\text{-}1}$ should be observed in both the *cc*- and *aa*-polarized spectra, as required by group theory.

S1 M.J. Frisch, G.W. Trucks, H.B. Schlegel, G.E. Scuseria, M.A. Robb, J.R. Cheeseman, G. Scalmani, V. Barone, B. Mennucci, G.A. Petersson, H. Nakatsuji, M. Caricato, X. Li, H.P. Hratchian, J.B. A. F. Izmaylov, G. Zheng, J. L. Sonnenberg, M. Hada, K.T. M. Ehara, R. Fukuda, J. Hasegawa, M. Ishida, T. Nakajima, O.K. Y. Honda, H. Nakai, T. Vreven, J. A. Montgomery, Jr., F.O. J. E. Peralta, M. Bearpark, J. J. Heyd, E. Brothers, V.N.S. K. N. Kudin, T. Keith, R. Kobayashi, J. Normand, A.R. K. Raghavachari, J. C. Burant, S. S. Iyengar, J. Tomasi, N.R. M. Cossi, J. M. Millam, M. Klene, J. E. Knox, J. B. Cross, C.A. V. Bakken, J. Jaramillo, R. Gomperts, R. E. Stratmann, A.J.A. O. Yazyev, R. Cammi, C. Pomelli, J. W. Ochterski, K.M. R. L. Martin, V. G. Zakrzewski, G. A. Voth, J.J.D. P. Salvador, S. Dapprich, A. D. Daniels, J.B.F. O. Farkas, J. V. Ortiz, J. Cioslowski and a.D.J. Fox, Gaussian Inc. (2010).

S2 Maksimuk, M., Yakushi, K., Taniguchi, H., Kanoda, K., & Kawamoto, A., The C=C Stretching Vibrations of κ-(BEDT–TTF)$_2$Cu[N(CN)$_2$]Br and Its Isotope Analogues, *J. Phys. Soc. Jpn.*, **70**, 3728 (2001).

S3 Yamamoto, T., Uruichi, M., Yamamoto, K., Yakushi, K., Kawamoto, A. & Taniguchi, H., Examination of the Charge-Sensitive Vibrational Modes in Bis(ethylenedithio)tetrathiafulvalene, *J. Phys. Chem. B*, **109**, 15226 (2005).

S4 Kini, A. M., Geiser, U., Wang, H. H., Carlson, K. D., Williams, J. M., Kwok, W., Vandervoort, K., Thompson, J. E., & Stupka, D. L., A new ambient-pressure organic superconductor, kappa-(ET)$_2$Cu[N(CN)2]Br, with the highest transition temperature yet observed (inductive onset Tc = 11.6 K, resistive onset = 12.5 K), *Inorg. Chem.*, **29**, 2555 (1990).
